# Contributing factors to high prevalence of hearing impairment in the Elias Motsoaledi Local Municipal area, South Africa: A rural perspective

**DOI:** 10.4102/sajcd.v66i1.611

**Published:** 2019-02-20

**Authors:** Karin Joubert, Donna Botha

**Affiliations:** 1 Department of Speech Pathology and Audiology, University of the Witwatersrand, South Africa; 2 Ndlovu Wits Audiology Clinic and Outreach Programme, South Africa; 3 Private Practice, Edenvale, South Africa

## Abstract

**Background:**

There is evidence that the factors contributing to the prevalence and aetiology of hearing impairment vary widely from one country to another. In South Africa, as in other low-income and middle-income countries, more context-specific information on the estimated prevalence of hearing impairment and the factors that contribute to its onset is required.

**Aim:**

The aim of this study was to provide decision-makers and hearing health professionals with local and accurate information on the prevalence of ear and hearing disorders in the Elias Motsoaledi Local Municipal (EMLM) area of the Limpopo province, South Africa.

**Methods:**

The World Health Organization (WHO) protocol for population-based surveys of prevalence and causes of deafness, hearing impairment and other ear diseases was utilised. A random multi-stage cluster sampling strategy, two-stage sampling, was utilised to select the seven municipal wards and 357 households through the probability proportional to size method. A total of 850 participants were included in the study.

**Results:**

The overall prevalence of hearing impairment was 19.88% (95% confidence interval [CI]: 0.15–0.2) and 8.94 (95% CI: 0.08–0.12) for disabling hearing impairment. The prevalence of ear disease was 13.19% (95% CI: 0.10–0.15), with impacted cerumen and otitis media reported most often. Associations with hearing impairment were established for age, gender and hypertension.

**Conclusion:**

The study has shown a higher prevalence of disabling hearing impairment in the rural EMLM area of the Limpopo province compared to global prevalence rates. In addition, known factors associated with hearing impairment were confirmed.

## Background

The devastating consequences of disabling hearing impairment^1^ for interpersonal communication, psychosocial well-being, economic independence and quality of life, irrespective of age of onset, are well documented in the literature (Olusanya, Neumann, & Saunders, 2014; Wilson, Tucci, Merson, & O’Donoghue, 2017). Global estimates indicate that the prevalence of disabling hearing impairment has increased from 5.73% in 2005 to 6.12% in 2018 (WHO, 2018). The years lived with disability (YLD) are high, as for disabling hearing impairment, the estimated YLD was 4.49% of the total YLDs because of all causes in 2015. Hearing impairment, now ranked as the fourth leading contributor of YLD, has thus become a major global health concern (Wilson et al., 2017).

Several factors contribute to the increased global prevalence estimates of disabling hearing impairment. Most notable is the significant association between hearing impairment and advanced age, which is attributed to increased mean life expectancy in many countries (GBD, 2016; Lin, Thorpe, Gordon-Salant, & Ferrucci, 2011; Olusanya et al., 2014; World Health Organization [WHO], 2018; Wilson et al., 2017). The improvement in the technology used for the early detection of hearing loss as well as the widespread use of ototoxic medications (e.g. for treatment of cancer, human immunodeficiency virus [HIV] and tuberculosis) are further cited as contributing factors (WHO, 2018; Wilson et al., 2017). Exposure to damagingly loud sounds (e.g. via occupational and recreational noise exposure) is also a growing concern (WHO, 2018). Other risk factors for hearing impairment (from prenatal through to adulthood) are outlined by Olusanya et al. (2014).

Approximately 50% of hearing impairment can be prevented and most of the remainder can be treated successfully (WHO, 2017; Wilson et al., 2017). Depending on the cause of hearing impairment, the treatment may include surgical and medical management, hearing aids, cochlear implants and other assistive listening devices. To prevent the onset of disabling hearing impairment and mitigate the impact of non-preventable hearing impairment (such as presbycusis) on the individual, family and society at large, improvements in the prevention thereof are imperative. The causes of hearing impairment, however, vary widely from one area to another (e.g. district, province and country) (Wilson et al., 2017). Prevention can thus only be effective if there is thorough understanding of the prevalence and aetiology of hearing impairment in a specific population.

The available estimates of the prevalence of hearing impairment and causes thereof in low-income and middle-income countries are limited (WHO, 2018), despite the fact that more than 80% of the people with disabling hearing impairment live in these countries (WHO, 2012). The findings of WHO ear and hearing surveys conducted in a variety of developing countries at provincial or subnational levels indicate that the prevalence of disabling hearing impairment ranges from 2.07% to 9% (Pascolini & Smith, 2009). In South Africa, a middle-income country, the estimated prevalence of hearing impairment is based primarily on information obtained from the national census data (Ramma & Sebothoma, 2016). Hearing impairment is the third highest reported disability after visual impairment and physical disability in South Africa (Statistics South Africa, 2012). Additional information obtained regarding the prevalence and causes of hearing impairment across the lifespan is available; however, the majority of these small-scale studies were conducted in urban areas and/or within the private healthcare sector (Joubert, Sebothoma, & Kgare, 2017). One of the first population-based surveys conducted in Cape Town, an urban metropolitan area in South Africa, reported a 4.57% estimated prevalence of disabling hearing impairment (Ramma & Sebothoma, 2016).

To reduce the burden of hearing impairment, the implementation of context-specific and targeted prevention programmes is vital. However, limited information is available on the prevalence and causes of hearing impairment as well as factors that contribute to the prevalence of thereof in the underserved, rural areas of South Africa (Joubert et al., 2017). The aim of this study was thus to provide decision-makers and hearing health professionals with local and accurate information on the prevalence of ear and hearing disorders in the Elias Motsoaledi Local Municipal (EMLM) area of the Limpopo province, South Africa. The objectives were to determine the prevalence of and factors associated with hearing impairment in this area.

## Methods

A cross-sectional population survey design was used to determine the prevalence of hearing impairment and auditory pathology in the EMLM area of the Limpopo province. The Ear and Hearing Disorders Survey Protocol (WHO, 1999) and an adaption of the WHO Ear and Hearing Disorders Examination Form–Version 8.3 (Appendix 1) were used to generate standardised data which allow for comparison among surveys conducted in different countries and regions (Ullauri et al., 2014). This form includes five areas: demographic information, hearing examination, basic ear assessment, cause of ear disease or hearing impairment and action needed.

### Context

The EMLM area located in the southern part of the Limpopo province forms part of the Sekhukhune District, the second poorest district in South Africa (Health Systems Trust, 2017). The EMLM has 30 municipal wards with an estimated population of 249 363 residing in 60 200 households with an average household size of four individuals (Statistics South Africa, 2012). This predominantly rural area is characterised by poor infrastructure, lack of safe water supply, high unemployment rates and low education levels (Health Systems Trust, 2017).

### Participants

#### Sampling strategy

A random multi-stage cluster sampling strategy, two-stage sampling, was utilised to select the clusters (municipal wards) through the probability proportional to size (PPS) method. The first stage entailed the random selection of seven clusters using a rural sampling frame. This resulted in the exclusion of five wards (e.g. those where the two major towns were situated and those with a large number of residents). The second stage involved the selection of households by dividing the total number of households in the five selected wards by the total number of households to be tested. This indicated that every 35th household in each of the selected wards was to be included in the survey. The total number of households in each selected ward was divided by 35 in order to determine how many households to visit in each ward. In cases where the individuals in the selected households declined participation, the research team visited the household to the left of the household that refused.

#### Sample size

The required sample size of 1428 participants was based on the 10% estimated prevalence of disabling hearing impairment with precision of 3.4% (confidence interval [CI]: 95%) (Oishi & Schacht, 2011). The final sample size included 357 households and 850 individuals from the selected households (59% of the proposed sample size). All individuals living in the selected households who gave consent and/or assent to participate were included in the study. The smaller than anticipated sample size can be attributed to the absence of many individuals (mostly men) on the day of the visit. These individuals were reportedly elsewhere because of work commitments. Only a small number of participants refused to participate in the study, citing time constraints and prior commitments as the reasons for refusal.

#### Participant description

The majority of participants (54.71%, *n* = 465) were between 15 and 64.11 years of age (Table 1). The unequal gender distribution could be attributed to the fact that in rural communities, men are more likely to work in more urban areas where the likelihood of finding employment is higher and as migrant workers, only returning home once a month, over long weekends or holidays (Food and Agriculture Organisation, 2014).

**TABLE 1:** Distribution of participants by age and gender (*n* = 850).

Age groups (years)	Male		Female		Total	
	*N*	%	*N*	%	*N*	%
0–3.11	41	12.28	22	4.26	63	7.41
4–14.11	128	38.32	123	23.84	251	29.53
15–64.11	148	44.31	317	61.43	465	54.71
>65	17	5.09	54	10.47	71	8.35

**Total**	**334**	**100**	**516**	**100**	**850**	**100**

### Research team

The research team consisted of four individuals: a qualified audiologist with 2 years’ clinical experience in the public healthcare sector and three research assistants. The research assistants were members from the study community and fluent speakers of the indigenous languages spoken in the area. The research assistants all completed Grade 12, and had previous experience in interpreting and conducting research through their involvement with a non-governmental organisation. Two of the research assistants had previous training and experience in performing hearing screening prior to their participation in the data collection for the current study. The research assistants received comprehensive training prior to the commencement of the study on the purpose of the study, how to approach households, obtaining informed consent, biologic calibration and use of equipment, accurate completion of the data collection forms, infection control and reviewing the maps of the areas where data collection took place. All research assistants signed confidentiality agreements after training and prior to data collection.

### Pilot study

A pilot study was conducted in a randomly selected ward in the same area to refine the study protocol. Households were selected as per the sampling strategy and potential participants informed about the study in their home language. Forty participants from nine households (average household size was 4.4 individuals) were included in the pilot study after giving written consent and/or assent. The same steps as outlined in the main study were followed and the study protocol completed with each participant. Data collected from these participants were not included in the main study. The majority of participants were female (65%; *n* = 26). The mean age of pilot study participants was 17.6 years (1 month – 62.4 years; ±19.98). Following the pilot study, the role of the research assistants expanded to include pure tone audiometry and automated auditory brainstem response (AABR) screening (with the interpretation of testing findings made by the researcher). In addition, the protocol for entry into the households was changed to first approach the house to explain the purpose of the study and to determine equipment needs (based on the ages of individuals in the household). This was more practical as the houses were too small to accommodate the research team and all the equipment.

### Assessment procedures and equipment

Average ambient noise level measured during testing was 55.53 dBA (±2.5) (Bruel & Kjaer 2239 Type II integrating sound-level meter). No statistically significant relationship between the level of ambient noise during testing and different degrees of hearing impairment was found (independent samples *t*-test).

Once pertinent case history information was obtained, an otoscopic examination (Welch Allyn otoscope) was conducted. The study protocol for participants younger than 4 years included distortion product otoacoustic emissions (DPOAEs) (OtoRead), tympanometry (Titan) and AABR (Maico MB11). For participants older than 4 years, tympanometry (Titan) and pure tone audiometry (AS608 screening audiometer) were conducted. The screening results were recorded on the ear and hearing disorders survey form.

### Data collection procedures

Once ethical clearance for the research study was obtained from the Human Research Ethics Committee (Medical) of the University of the Witwatersrand (Protocol number: M130238) and permission granted by the Limpopo Department of Health and Social Development, data collection commenced.

Community entry was made through permission sought from ward councillors and tribal authorities (e.g. village chiefs) prior to entry into the selected wards. The community was informed of the study by announcing the research project on Moutse FM, the local radio station.

The research team visited each selected household and if permission was granted to enter the household, each individual was presented with the participant information sheet (if age appropriate) and the study was verbally explained to them. The participants were fully informed of the nature of the study and assured of confidentiality and their rights to withdraw from the study at any time without negative consequences. Only participants who gave written consent and/or assent were included in the study.

The audiometric assessment was conducted as per the study protocol and participants who required ear and hearing health care were referred to their nearest health facility. All completed survey forms were verified for completeness by the audiologist and data transferred onto an Excel spreadsheet by the audiologist.

### Reliability and validity

Non-random errors were minimised as research assistants received comprehensive training on the study protocol under field conditions and had access to the comprehensive training manual throughout the study. The routine audiological test procedures used in the study are all valid measures with high sensitivity and specificity. All the equipment used in the study was calibrated prior to the start of the study (SABS 0154-1; 0154-2). Daily listening checks and biological calibration were performed before each data collection session. The audiologist accompanied the research assistants on all visits and completed the same data collection forms as the assistants to determine the inter-rater reliability. Inter-rater agreement was 100%.

### Data analysis

SAS Version 9.3, a statistical software programme, was used to conduct data analysis. Descriptive statistics used included measures of central tendency and variability. An independent *t*-test (*p* = 0.05) was used to determine the relationship between ambient noise levels and hearing thresholds obtained. Binomial proportion confidence intervals (*p* = 0.05) were used to estimate the probability (odds) of hearing impairment. The relationship of various factors associated with hearing impairment was analysed with binomial logistic regression (*p* = 0.05).

### Ethical consideration

Approval was granted for this study with ethical clearance number: M130238.

## Results

### Prevalence of hearing impairment

The prevalence of hearing impairment (>26 dB) in this population was 19.88% (95% CI: 0.15–0.21) (Table 2).

**TABLE 2:** Proportion of participants with hearing impairment (%) according to gender and World Health Organization age group categories (*n* = 191).

Age group (years)	Male		Female		Total	
	%	CI	%	CI	%	CI
0–14.11	13.61	0.09–0.17	14.48	0.08–0.22	14.01	0.12–0.21
15–64.11	16.22	0.12–0.27	22.08	0.21–0.31	20.22	0.14–0.23
>65	70.59	0.47–0.90	75.93	0.62–0.85	74.65	0.62–0.83

**Total**	**17.66**	**0.16–0.26**	**25.58**	**0.21–0.28**	**19.88**	**0.15–0.21**

CI, confidence interval.

With the specific classification of hearing loss into various degrees, the prevalence of slight hearing impairment was 10.94% and that of disabling hearing impairment was 8.94% (Table 3). The prevalence of moderate hearing impairment was 5.88%; for severe impairment it was 2.24% and for profound hearing impairment it was 0.82%. As expected, the highest prevalence of disabling hearing impairment at 46.48% (95% CI: 0.36–0.53) was found in the >65 year age group.

**TABLE 3:** Proportion of participants with hearing impairment (%) per age group (*n* = 850).

Age group (years)	*n*	Slight impairment† (26 dB – 40 dB)‡		DHI (>40 dB)‡	
		%	CI	%	CI
0–3.11	63	0	0	1.91	0.01–0.05
4–14.11	251	6.77	0.04–0.11	1.91	0.01–0.05
15–64.11	465	12.04	0.1–0.17	7.96	0.06–0.11
>65	71	28.17	0.26–0.53	46.48	0.36–0.53

**Total**	**850**	**10.94**	**0.09–0.14**	**8.94**	**0.08–0.12**

DHI, Disabling hearing impairment.

† , World Health Organization (WHO) criteria for grading of hearing impairment;

‡ , Grading is according to the WHO. The degree of hearing loss is indicated in brackets. Hearing loss >40 dB in the better hearing ear in adults and >30 dB in children.

Because of the nature of the screening measures (DPOAE and AABR) used for children 0–3.11 years of age, it was not possible to differentiate between the degrees of hearing impairment. Instead, results were recorded as either ‘pass’ or ‘refer’. A ‘pass’ result indicated that they presented with normal middle ear and outer hair cell functioning. Only 37.5% (*n* = 21) of the children in this age group obtained a ‘refer’ result. The majority of these participants were males (57.2%; *n* = 12).

### Factors associated with hearing impairment

The cause of ear disease and/or hearing impairment was reported as per the WHO classification (WHO, 1999). The prevalence of ear disease in this population was 13.19% (95% CI: 0.10–0.15), with impacted cerumen as the most prevalent (10%), followed by otitis media (2.48%) (Table 4).

**TABLE 4:** Types of ear disease according to gender and World Health Organization age group categories (*n* = 112).

Age group (years)	Gender	Cerumen		Otitis media		Foreign bodies		Total	
		*n*	%	*n*	%	*n*	%	*n*	%
0–3	Male	1	0.12	6	0.71	0	0	7	0.82
	Female	1	0.12	3	0.35	0	0	4	0.47
	Total	2	0.24	9	1.06	0	0	11	1.30
4–14.11	Male	19	2.24	2	0.24	1	0.12	22	2.6
	Female	20	2.35	6	0.71	3	0.35	29	3.41
	Total	39	4.59	8	0.95	4	0.47	51	6.01
15–64.11	Male	17	2	3	0.35	0	0	20	2.35
	Female	20	2.35	1	0.12	1	0.12	22	2.59
	Total	37	4.35	4	0.47	1	0.12	42	4.94
>65	Male	0	0	0	0	0	0	0	0
	Female	7	0.82	0	0	1	0.12	8	0.94
	Total	7	0.82	0	0	1	0.12	8	0.94

**Total**	**-**	**85**	**10**	**21**	**2.48**	**6**	**0.71**	**112**	**13.19**

Of the participants who presented with disabling hearing impairment (*n* = 169), the majority (66%; *n* = 112) reported that the cause of their hearing impairment was unknown (undetermined).

**TABLE 5:** Factors associated with disabling hearing impairment.

Factors	Odds ratio	95% CI	*p*
Age	14.86	8.50–25.97	<0.0001*
Gender	1.78	1.05–2.99	0.0309*
Hypertension (self-reported)	9.97	5.90–16.85	<0.0001*
Use of ototoxic medication	2.24	0.82–6.15	0.1169

CI, confidence interval.

* , *p* = 0.05.

The association between hearing impairment and age, gender, hypertension and use of potentially ototoxic medication (for cancer, tuberculosis and self-reported HIV) was determined. The findings of the current study indicate that age and self-reported hypertension were associated with hearing impairment (Table 5).

### Ear and hearing care services required

Thirty-one per cent (*n* = 261) of the participants required referrals to the hospital or primary healthcare clinic for further management. Whilst the overwhelming majority of these individuals (68%; *n* = 178) required diagnostic audiometry to confirm the type and degree of hearing impairment, cerumen management was required for 35% (*n* = 92) and medical management was required for 10% (*n* = 26) of these participants.

## Discussion

A key finding of this study is that the prevalence of disabling hearing impairment in this rural population (8.94%) is higher than the reported global estimate of 6.12% (WHO, 2018) and other developing countries where the prevalence rates ranged, for example, from 4.7% (in Southern Vietnam) to 7.6% in Nigeria (WHO, 2012). Most notable is the fact that the prevalence rate found in the current study is significantly higher (*p* < 0.0001) than the estimated prevalence rate (4.57%) reported for the Cape Town metropolitan area, South Africa (Ramma & Sebothoma, 2016).

The high prevalence of ear disease reported in the current study was mainly because of impacted cerumen and otitis media. Globally, the prevalence of impacted cerumen varies widely. In the United Kingdom, between 2% and 6% of the general population present with cerumen impaction at any given time, whilst the diagnoses of 3.6% of emergency visits for otologic complaints in the United States were impacted cerumen (Schwartz et al., 2017). The reported prevalence of impacted cerumen (10%) in the current study is similar to the findings of a population-based survey conducted in Brazil, which was between 8.4% and 13.7% (Ullauri et al., 2014). Hearing loss as a result of cerumen occlusion can range from 5 dB to 40 dB depending on the degree of canal occlusion (Schwartz et al., 2017).

Although otitis media is a common condition, only 2.48% of the population in this rural area presented with otitis media as confirmed with otoscopy and tympanometry. This rate is lower if compared to the rates reported globally (11%) (WHO, 2018) as well as in other developing countries such as Sri Lanka (9%), Myanmar (8%), Brazil (6.8%), Vietnam (5.99%) and India (6%) (Beria et al., 2007). Residents in the rural community studied were not exposed to the typical high risks for otitis media, such as living in overcrowded households (average household size is 4.4) and lack of hygiene (Copley & Friderichs, 2010).

Various factors are reported to contribute to the increased global prevalence of disabling hearing impairment (GBD, 2016; Lin et al., 2011; Olusanya et al., 2014; WHO, 2018; Wilson et al., 2017; Ramma & Sebothoma, 2016). In the current study, the main factors associated with disabling hearing impairment were age, gender and a self-reported history of hypertension. The significant association between disabling hearing impairment and advanced age is well reported (Lin et al., 2011; WHO, 2018; Wilson et al., 2017). Although age-related hearing impairment is not preventable, the impact thereof on interpersonal communication, psychosocial well-being and quality of life can be mitigated by early identification and appropriate management. The current study further supported the hypothesis that there is an association between cardiovascular disease and disabling hearing impairment (Ramma & Sebothoma, 2016; Rosenhall & Sundh, 2006; Solanki, 2012). A systematic review of the literature correlated cardiovascular disease to low-frequency hearing loss, especially in women (Rosenhall & Sundh, 2006). A recent South African study reported low-frequency hearing impairment in 5% of individuals between the ages of 40 and 55 years (*n* = 92) diagnosed with cardiovascular disease (Solanki, 2012).

In addition to the factors already discussed, the limited availability of comprehensive ear and hearing care programmes and services is postulated to be a major contributing factor to the high prevalence of disabling hearing impairment in the study population.

This study is, to the best of our knowledge, the first study to determine the prevalence of disabling hearing loss in a rural area of South Africa. The use of the standardised WHO Ear and Hearing Disorders Survey (WHO, 1999) allowed for comparisons to be made to other contexts: locally, nationally and globally. The study also has limitations. Firstly, as this was a cross-sectional study, it is not possible to establish true cause and effect relationships based on the data. Secondly, the use of self-reported diagnosis of cardiovascular disease and HIV may have resulted in an underestimation of the prevalence of these diseases and its effect on hearing. Future research to determine the prevalence of ototoxic and noise-induced hearing loss in developing countries as well as the effectiveness of targeted, context-specific ear and hearing care services should be conducted.

In summary, our data have shown a higher prevalence of disabling hearing impairment in the rural EMLM area of the Limpopo province compared to global prevalence rates. In addition, known factors associated with hearing impairment (age, gender and cardiovascular disease) and ear disease (cerumen impaction and otitis media) were confirmed. Although ototoxicity and noise-induced hearing loss were not associated with hearing impairment in this population, the importance thereof in causing hearing impairment should not be negated.

## Conclusion

The study was successful in providing the decision-makers in the national and provincial departments of health as well as hearing health professionals with information on the prevalence and causes of ear and hearing disorders in this rural area. The findings can be used to plan for and implement more accessible ear and hearing care services that are specifically tailored to the needs identified. The strategies outlined by Olusanya et al. (2014) provide a road map for the prevention of hearing loss that can be tailor-made for this community. They suggest that prevention can be carried out at three levels – primary, secondary and tertiary levels – and provide examples of specific activities that can be implemented across the lifespan. Following the implementation of improved and more accessible ear and hearing care services in the EMLM area, the findings of this study will serve as a baseline for future surveillance studies to monitor performance and determine impact.

## Figures and Tables

**FIGURE 1-A1:**
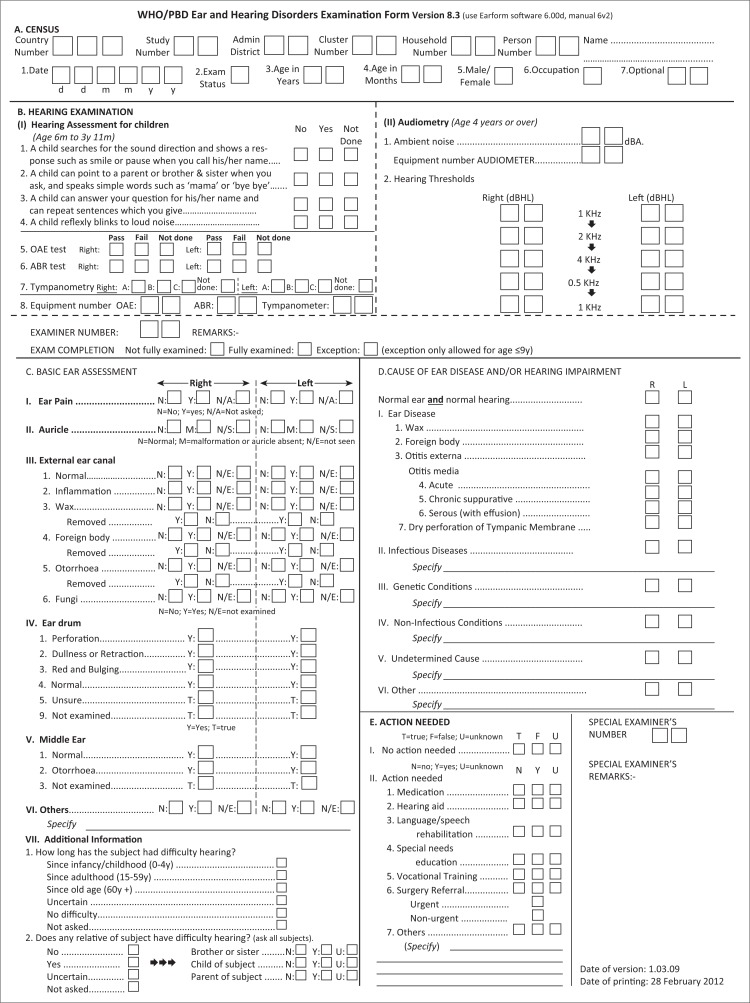
World Health Organization Ear and Hearing Disorders Examination Form: Version 8.3.

## Footnotes

1 Disabling hearing impairment: Hearing loss > 40 dB in the better hearing ear in adults and > 30 dB in children.
